# Editorial: Mindfulness in internet and new media

**DOI:** 10.3389/fpsyg.2023.1233809

**Published:** 2023-09-22

**Authors:** Chao Liu, Hao Chen, Jing-Wen Zhuo, Wen-Ko Chiou

**Affiliations:** ^1^School of Journalism and Communication, Hua Qiao University, Xiamen, China; ^2^School of Film Television and Communication, Xiamen University of Technology, Xiamen, China; ^3^Department of Industrial Engineering and Management, Ming Chi University of Technology, New Taipei, Taiwan; ^4^Department of Psychiatry, Chang Gung Memorial Hospital, Taoyuan, Taiwan; ^5^Department of Industrial Design, Chang Gung University, Taoyuan, Taiwan

**Keywords:** mindfulness, meditation, internet, new media, loving kindness meditation (LKM)

In the rapidly evolving landscape of the digital age, our relationship with the internet and new media has become more intertwined than ever before. We find ourselves immersed in a virtual world, constantly bombarded by information, and connected to a global network of individuals. This Research Topic, “*Mindfulness in internet and new media*,” explores the complex interplay between our digital lives and the practice of mindfulness.

Mindfulness, a mental state characterized by awareness and presence in the moment, has gained increasing attention in recent years for its potential to enhance wellbeing and cognitive functioning. As we navigate the ever-expanding digital realm, questions arise about how mindfulness can be applied to foster a more balanced and harmonious relationship with the internet and new media.

## The evolution of digital mindfulness

The roots of mindfulness trace back to ancient contemplative practices, but in today's context, it has taken on new dimensions. In this Research Topic, we delve into how mindfulness has adapted and evolved to meet the challenges of the digital age. Our contributors explore various aspects of digital mindfulness, ranging from its impact on mental health to its role in cultivating meaningful connections in online communities.

## Articles in this Research Topic

The first article in this Research Topic, “*The effect of animation-guided mindfulness meditation on the promotion of creativity, flow, and affect*,” by Chen et al., explores the innovative use of animation in mindfulness practices. This research investigates how animation can enhance the effectiveness of mindfulness meditation, fostering creativity and emotional wellbeing. The study's findings provide insights into the potential for technology to augment traditional mindfulness practices (Chen et al.).

In “*Effects of online mindfulness-based interventions on the mental health of university students: A systematic review and meta-analysis*,” Gong et al. conduct a comprehensive analysis of existing research in the field. This article synthesizes the current state of knowledge regarding the impact of online mindfulness interventions on the mental health of university students. The findings have implications for educators and mental health professionals working with the student population in digital spaces (Gong et al.).

Gu et al. delves into the world of social media in “*The effect of trait mindfulness on social media rumination: Upward social comparison as a moderated mediator*.” This study explores how individual differences in mindfulness can influence our online behaviors, specifically focusing on the phenomenon of social media rumination. By highlighting the role of upward social comparison, the research provides insights into the dynamics of social media use (Gu et al.).

The article titled “*A study on the relationship between mindfulness and work performance of web editors: Based on the chain mediating effect of workplace spirituality and digital competencies*” by He et al. take a workplace perspective. It investigates how mindfulness practices can enhance the performance of web editors. The study introduces the concept of workplace spirituality and digital competencies as key mediators, offering a holistic view of the relationship between mindfulness and work outcomes in the digital age (He et al.).

“*Techno-psychological approach to understanding problematic use of short-form video applications: the role of flow*” by Huang et al. offer a perspective on problematic technology use. Focusing on short-form video applications, this research explores the concept of flow as a driver of excessive engagement. Understanding the role of flow in technology addiction is crucial for developing interventions and strategies to promote healthy digital behaviors (Huang et al.).

Kuang et al. examine the impact of mindfulness in the workplace in “*The effect of employee mindfulness in the new media industry on innovative behavior: The chain mediating role of positive emotion and work engagement*.” This study highlights how mindfulness practices can enhance innovative behavior among employees in the new media sector, emphasizing the importance of positive emotions and work engagement in this process (Kuang et al.).

In “*Effects of animated pedagogical agent-guided loving-kindness meditation on flight attendants' spirituality, mindfulness, subjective wellbeing, and social presence*,” Liu et al. explore the use of mindfulness in the airline industry. This research introduces animated pedagogical agents to guide meditation practices, demonstrating how technology can facilitate mindfulness and enhance wellbeing in specific occupational settings (Liu et al.).

“*Parental intervention strategies and operating mechanism on adolescent social media use—The concept of literacy improvement based on interaction*” by Wang and Chen focus on the critical issue of adolescent social media use. This article discusses parental intervention strategies and the concept of literacy improvement through interaction. Understanding how parents can guide their children's digital interactions is crucial for promoting responsible internet use among youth (Wang and Chen).

Wang et al. address the design of mindfulness information in “*Designing mindfulness information for interaction in social media: The role of information framing, health risk perception, and lay theories of health*.” This research investigates how the presentation of mindfulness content can influence user engagement and health perceptions on social media platforms. It sheds light on the design principles that can make mindfulness information more effective in digital contexts (Wang et al.).

“*The effect of mindfulness intervention on internet negative news perception and processing: An implicit and explicit approach*” by Yang et al. delves into the impact of mindfulness on our consumption of online news. This study employs both implicit and explicit measures to understand how mindfulness interventions can influence our perception and processing of negative news, offering novel insights into the intersection of mindfulness and media consumption (Yang et al.).

You and Liu investigate the relationship between mindfulness and online behaviors in “*The effect of mindfulness on online self-presentation, pressure, and addiction on social media*.” This article explores how mindfulness can mitigate the pressures associated with online self-presentation and addiction tendencies on social media platforms. It underscores the potential for mindfulness to promote healthier digital interactions (You and Liu).

Finally, “*A study of the factors influencing HIV-preventive intentions among 'hookup' application users*” by Li and Li explore the complex world of dating and hookup applications. This research examines the factors that influence HIV-preventive intentions among users of these apps, shedding light on the intersection of technology, sexual health, and mindfulness (Li and Li).

## The significance of mindfulness in the digital age

Our contributors have illuminated various dimensions of mindfulness in the context of internet and new media. Together, their work underscores the significance of cultivating mindfulness in an era dominated by screens and constant connectivity.

Collectively, the articles in this Research Topic offer a comprehensive exploration of mindfulness in the digital age. They underscore the significance of mindfulness in helping us navigate the complexities and challenges of the internet and new media. Here are some key takeaways: enhance creativity and emotional wellbeing, improve student mental health, understand social media behavior, improve work performance, change problematic media use behaviors, foster innovation, enhance the wellbeing, guide parent education, guide interaction design, perception, and processing of negative news, online self-presentation and addiction and sexual health decisions.

## Conclusion

As we conclude this expansive Research Topic on “*Mindfulness in internet and new media*,” we extend our heartfelt gratitude to the authors, reviewers, and readers who have contributed to this comprehensive exploration of the intersection between mindfulness and the digital landscape. The research presented here underscores the vital role that mindfulness plays in helping us navigate the complexities and challenges of the internet and new media. It offers a roadmap for cultivating a more balanced, mindful, and ethical engagement with the digital world.

In an era where the internet and new media are integral to our daily existence, the practice of mindfulness becomes more than just a tool for personal wellbeing—it becomes a means to navigate the digital landscape with clarity, balance, and intention.

## Author contributions

All authors listed have made a substantial, direct, and intellectual contribution to the work and approved it for publication.

